# Impact of Compression and Small Cell Deployment on NB-IoT Devices Coverage and Energy Consumption with a Realistic Simulation Model

**DOI:** 10.3390/s21196534

**Published:** 2021-09-30

**Authors:** Mehdi Zeinali, John S. Thompson

**Affiliations:** 1 Digital Innovation Research Group, Department of Engineering, Nottingham Trent University, Nottingham NG11 8NS, UK; 2 Institute for Digital Communications, Alexander Graham Bell Building, School of Engineering and Electronics, Kings Buildings, Mayfield Road, Edinburgh EH9 3JL, UK; j.s.thompson@ed.ac.uk

**Keywords:** compression, small cell, NB-IoT, energy consumption modeling, huffman, Lempel-Ziv-Welch, latency, LPWAN

## Abstract

In the last few years, Low-Power Wide-Area Network (LPWAN) technologies have been proposed for Machine-Type Communications (MTC). In this paper, we evaluate wireless relay technologies that can improve LPWAN coverage for smart meter communication applications. We provide a realistic coverage analysis using a realistic correlated shadow-fading map and path-loss calculation for the environment. Our analysis shows significant reductions in the number of MTC devices in outage by deploying either small cells or Device-to-Device (D2D) communications. In addition, we analyzed the energy consumption of the MTC devices for different data packet sizes and Maximum Coupling Loss (MCL) values. Finally, we study how compression techniques can extend the battery lifetime of MTC devices.

## 1. Introduction

Until recently, cellular communication technologies have been designed to support traffic for human communications called Human-Type Communication (HTC). It is worth saying that Machine-Type Communications (MTC) is distinct from HTC in terms of the data traffic pattern, required latency and deployment density [1]. The main parameters to be considered in MTC communications for the underlying radio technologies are low data rate, scalability, wide-area coverage and low power consumption. Considering such requirements, most of the well-known short-range communications systems such as Wi-Fi, ZigBee and Bluetooth low energy will not be applicable for the metering infrastructure. At the same time, long-range wireless cellular technologies such as third generation (3G) and fourth generation (4G) cannot easily be used in this context because of high energy consumption, the high cost of equipment and because they have been designed for high-speed human-centric communications.

Due to all these considerations, low-power wide-area networks (LPWAN) will be the most suitable option for smart metering in the context of the smart grid [2]. As LPWAN communication technologies have been standardized in the last few years, they are very attractive for both smart grid and wider internet of things (IoT) applications. Different properties and aspects of emerging LPWAN technologies have been discussed in these references [3,4] in more detail. The third-generation partnership project (3GPP) introduced its LPWAN solution, narrow-band IoT (NB-IoT), in its LTE Release 13 [5]. The use of NB-IoT technology [2] has been studied recently for smart metering or smart grid applications. Some comparative studies regarding deployment cost, latency, range and other aspects have been summarized in [4]. Simple energy consumption and throughput modeling of NB-IoT in comparison with general packet radio service (GPRS) technologies has been discussed in [6]. Compared to other LPWAN technologies, using NB-IoT is advantageous due to the low-cost chipset, better building penetration and lower power consumption due to the simpler waveform. Due to these advantages will be the best option for static IoT devices such as smart meters. In [7], a prototype system including NB-IoT devices, an IoT cloud platform, and an application server has been tested. Other important aspects of NB-IoT which needs to be addressed are the capacity and coverage which are discussed in [8]. Finally, in [9], the authors conducted NB-IoT network performance analysis in a real-world indoor environment. The small cell concept has been defined as low-power access points that operate in licensed spectrum to improve cellular coverage and capacity and can be deployed in homes and enterprises [3]. Small cells can enhance MTC device coverage and provide a backhaul link over an internet connection to the core network.

This paper significantly extends the initial research work in [3]. We have improved our simple evaluation in that paper using more realistic model for small cell propagation using a more precise path-loss models and using a realistic correlated shadow-fading map. Moreover, we also study device-to-device (D2D) communications to improve MTC device range. Furthermore, the energy consumption of the MTC devices using NB-IoT technology has been analyzed precisely, and compression techniques have been proposed to increase the lifetime of the battery. The remainder of this paper is structured as follows. Section 2 discusses system modeling and the communications scenario. Section 3 discusses simulation results for the impact of deploying small cells or D2D methods to improve coverage area for MTC devices. In addition, Energy consumption analysis is also given that considers the benefits of compression techniques. Section 4, discusses results from testbed for implementation of compression algorithms and impact of these algorithms on improving latency using wireless cellular technologies. Finally, Section 5 presents the paper conclusions.

## 2. System Modeling

NB-IoT has been designed specifically for IoT applications by 3GPP by modifying the basic functionalities of LTE. However, NB-IoT requires 20 dB more maximum coupling loss (MCL) for serving end node devices. Several LTE protocols have been modified to achieve this gain, such as new signaling and control channels for NB-IoT. Furthermore, LTE uses Frequency Division Duplexing (FDD) supporting full-duplex mode while NB-IoT uses the same techniques in half-duplex type-B. This reduces the complexity of the MTC device, but it means that it cannot transmit (uplink) and receive (downlink) data simultaneously [5].

Two important critical factors that need to be considered in designing wireless communication systems are achievable data rate and signal coverage. By defining key parameters, we can characterize the wireless communication channel. As a result, we can calculate the received signal quality using a propagation model for a given distance from the transmitter. The 3GPP standard path-loss model [10] has been used in this paper to model cellular IoT devices. Maximum Coupling Loss (MCL) or the communication link budget is used for simulation of downlink (DL) and uplink (UL) to identify the coverage issues. Different parameters such as receiver sensitivity, shadowing, path loss, etc. affect the attenuation between the eNodeB antenna ports and the MTC device, dictating a limiting value for the MCL. The required MCL value is 164 dB [5] for MTC devices in NB-IoT cellular networks and can be defined as:

(1) $$MCL\left(dB\right)=EIRP-L_{\mathrm{Total}\;}+G_{\mathrm{RX}}$$

where $G_{\mathrm{RX}}$ is the receiver antenna gain to fulfil the target signal threshold, *EIRP* is the effective isotropic radiated power which comprises the transmitter antenna gain plus transmitter power. Finally, $L_{\mathrm{Total}}$ includes effective noise power and all losses, including path loss.

**Figure 1:**
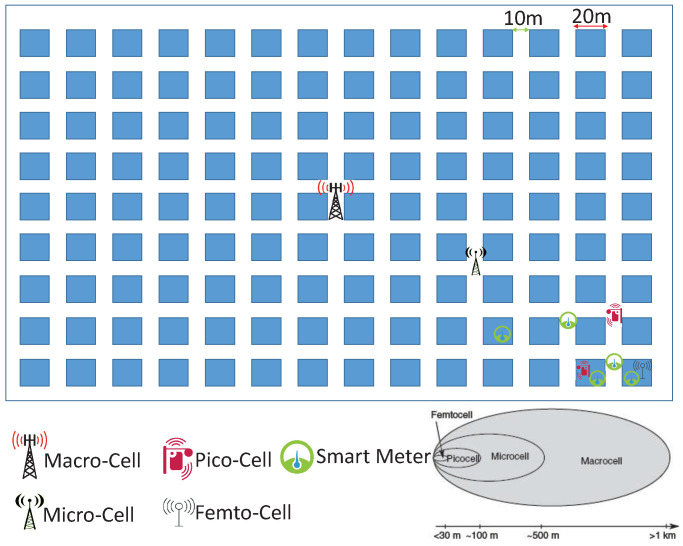
Small Cell Deployment Scenarios.

Our communications scenario is as can be seen in Figure 1. In this scenario we deployed small cells such as femto and pico-cells in the area covered by main macro-cell base station to improve the coverage for cellular IoT end-users in outage. We define a user in an outage when the user cannot communicate data, and the MCL calculated using Equation (1) is higher than that required for NB-IoT: therefore, a user in outage has an MCL > 164 dB. Then we can calculate the received signal power to the UE device from the BS using path-loss models presented in Equations (2)–(4). The study of cellular communications systems requires us to consider the main parameters such as multi-path fading, path loss and shadow fading, which can attenuate the wireless signal between the base station and end-user devices.

### 2.1. Path Loss

The large-scale path-loss model for the communication link between Base Station (BS) and user equipment (UE) according to Annex A of 3GPP standard [5] for the deployment scenario of Cellular IoT is as follows:

(2) $$\begin{array}{cc} \; & L_{\mathrm{BS}-\mathrm{UE}}=\;120.9+37.6\log_{10}\left(d\right)\left(\mathrm{dB}\right) \end{array}$$

where: *d* is the separation distance (km) between the base station and the user equipment and has been studied in [3]. In continue, we studied different standardized path-loss models in the international telecommunication union (ITU) and 3GPP documents to find the most suitable path-loss model, including the critical factors that affect signal attenuation. Finally, we chose the path-loss models shown in Equations (3) and (4) respectively from the ITU [11], and 3GPP [12] as both models provide an appropriate mathematical representation indoor pico-cell of radio propagation. The ITU basic path-loss model is:

(3) $$L_{\mathrm{pico}}=20\log_{10}f+N\log_{10}d+L_{f}\left(n\right)-28\left(\mathrm{dB}\right)$$

where: *d* is the separation distance (m) between the base station and the user equipment, *N* is distance power loss coefficient, *f* is the frequency (MHz), *n* is the number of floors between base and portable and $L_{f}$ is the floor penetration loss factor (dB). The values of *N* and $L_{f}$ for different frequencies has been given in [11]. The microcell propagation model has been obtained from 3GPP 36.814 standard [12], and it has the following form:

(4) $$\begin{array}{cc} \; & L_{\mathrm{micro}}=\max\left(38.46+20\log_{10}R_{2}+0.7d_{2D,\mathrm{indoor}}+L_{\mathrm{ow}},15.3+37.6\log_{10}R_{2}\right)\left(\mathrm{dB}\right) \end{array}$$

where $d_{2D,\mathrm{indoor}}$ is the distance inside the house, $R_{2}$ is the distance between receiver and transmitter, and finally $L_{\mathrm{ow}}$ is the penetration loss of one outdoor wall which is 10 dB.

In Figure 1 three possible propagation scenarios exist which are described as follows:

1. The user Equipment (UE) such as a smart meter is inside the same house as a small cell (Femto cell or Pico-cell) Base Station (BS);
2. The UE is outside of the building;
3. The UE is inside a different house which will add $L_{\mathrm{ow},1}$ and $L_{\mathrm{ow},2}$ to the path-loss model for the wall attenuation in buildings one and two, respectively.

The distances of UEs from different BSs is calculated on based on the scenario in Figure 1 for each building (20 m) and street (10 m) and the number of walls applied in the path-loss model. In this scenario we have assumed that femto cells (range of <30 m) are only installed inside building but for pico-cells (range of <100 m) we have possibility to install them both inside and outside.

### 2.2. Shadow Fading

**Figure 2:**
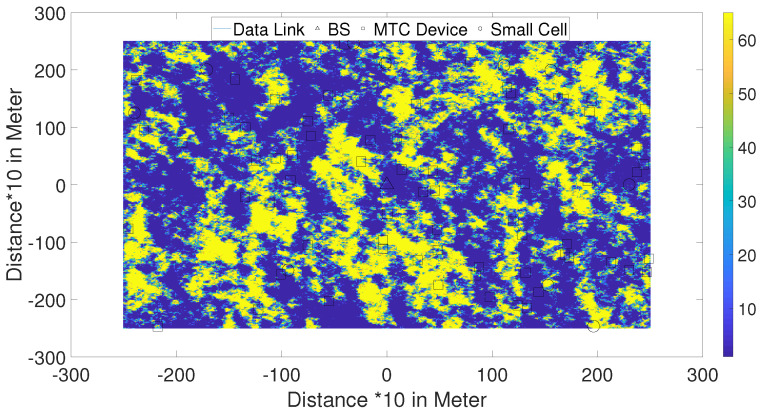
Applying Shadow Fading Map to our simulation analysis.

The final step to complete the small cell deployment in our model is to consider a realistic model for shadow fading [13]. In [3] we have considered shadow fading with a simple log-normal shadowing value but here we replaced that model with the method in [13] which generates a correlated shadow fading map. In the proposed model, correlated shadow fading can be described simply with normalized correlation function:

(5) $$r\left(x\right)=e^{-\alpha x},\;x\geq 0$$

where *x* is the distance and $e^{-\alpha }$ is the correlation coefficient between two UE locations spaced by 1 m using the suggested value of α=1/20. Using this value of α means that the shadow-fading correlation reduces to a value of 0.5 when the UEs are spaced by a distance *x* of approximately 14 m. In our simulation we therefore assumed the shadow fading is unchanged over a distance of 10 m and therefore we construct our map with square micro-cells with length of 10 m. Figure 2 shows one realization of a Monte Carlo simulation with a shadow-fading map integrated into the simulation scenario shown in Figure 1. To generate the correlated fading map in Figure 2, we used two-dimensional space using four neighbors (each neighbor and square of 10 m) to create correlation matrices as explained in the Appendix of [13]. In Figure 2 we have a large square map around a macro-cell base station at the center of map with length and width of 6 km. Based on the interpretation of the map, the value of shadow-fading attenuation can be calculated between the macro-cell base station and each UE and then used in the simulation of the coverage analysis.

Our Monte Carlo simulation steps can be summarized as follows:

- A shadow-fading map is created using the algorithm in the Appendix of [13]. The BS is located at the center of the map with randomly spread UE devices around the map as can be seen in Figure 2.
- Then the physical BS/UE locations in Figure 1 are mapped to the shadow-fading values shown in Figure 2. This process allows the simulation to identify where the UE has been located and if any walls are present in the BS-UE link that need to be accounted for in the path-loss calculation.
- According to the geometry of the UE and BS and the path-loss model, we measured the received signal power level in the location of the UE devices.
- Finally, we calculate the percentage of UE devices in outage. Then we implement the D2D communication scenario or add small cells randomly to the map. Again, we calculate the received signal level from nearby UE devices for the D2D communication scenario or the small cell assisted scenario. Using the data provided below in Section 3, the MCL can be computed for the D2D/small cell wireless links to identify the improvement of outage for UE devices.

### 2.3. NB-IoT Energy Consumption Modeling Based on 3GPP Standards

As shown in Table 1 [14], the total energy consumption can be broken down into four main blocks for an IoT device to operate: $E_{Communication}$ required energy to communicate the data, which is typically 60% of the total; $E_{\mathrm{Collection}}$ for collecting (6–20% of the total) and $E_{\mathrm{Processing}}$ (15–30%) for processing collected data. Furthermore, a small portion of the energy (1–6%), which we can call $E_{system}$, is consumed to wake up the machines periodically or run a real-time operating system. Summing all these energy terms together expresses the total consumed energy of the devices $E_{Device}$ as can be seen in the following expression:

(6) $$\begin{array}{c} E_{\mathrm{Device}}=E_{\mathrm{Communication}}+E_{\mathrm{Collection}}+E_{\mathrm{Processing}}+E_{\mathrm{System}} \end{array}$$

In order for the IoT device to perform the tasks, it needs to wake up each time and complete collection, processing, and communication of data in a particular time that we can call $T_{Recording}$. Additionally, $T_{\mathrm{messaging}}$ can be defined as the required time that IoT devices need to communicate the processed message.

The data processing and communications operations for the smart metering application can be split into three steps:

- Energy consumption measurement of each circuit in the building;
- Applying compression techniques to the collected data to reduce the size of data using smart meter hardware
- Updating the Energy Data Center (EDC) information by transmitting the compressed data or detecting an unusual situation to activate an alarm, by creating a data packet of duration Message $T_{messaging}$.

### 2.4. Network Power Consumption Modeling

Essential requirements such as lifetime, available energy, and reduced cost need to be considered in modeling IoT applications’ energy consumption. To the best of our knowledge, for modeling network energy consumption, there are two main scenarios:

1. Point-to-Point Communications (PPC) [15]
2. Time Synchronized Networks (TSN) [16]

**Figure 3:**
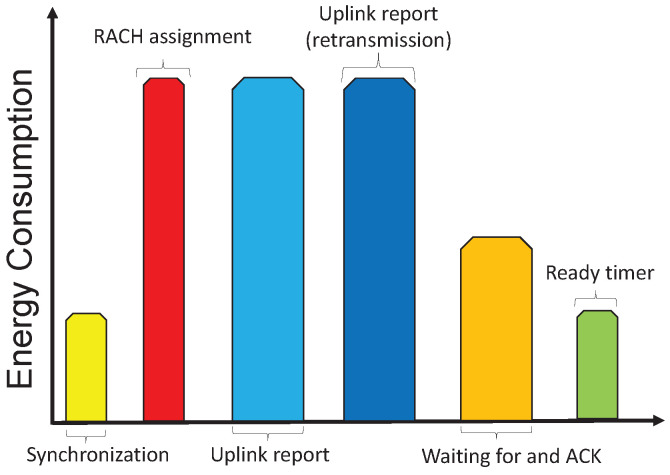
Protocol flow for the uplink of NB-IoT showing an example of possible energy consumption.

We have used the PPC model for our simulations, considering parameters such as interference-free and a single hop communication scenario. Additionally, in our simulation, we have assumed the Medium Access Control (MAC) layer is ideal. This assumption means that co-channel interference and packet collisions can be neglected, so that any transmitted data packet can be assumed to reach the receiver correctly. The energy consumption in this scenario should be calculated separately for each MTC device. According to Figure 3, the device should consume energy $E_{Datapacket}$ for the *K*th transmitted data packet, including all energy consumed in Figure 3 in a time of $T_{Datapacket}\left(K\right)$. As a result, for transmitting all the $N_{DataPacket}$ messages, the average power consumption can be expressed as:

(7) $$P_{\mathrm{Network}}=\left(1/N_{DataPacket}\right)\sum_{K=0}^{N_{DataPacket}}\frac{E_{Datapacket}}{T_{Datapacket}\left(K\right)}$$

For different wireless communication technologies, as shown in Equation (7), $E_{Datapacket}$ is a changing parameter. The two main terms of Equation (7) that can impact total energy consumption are the radio power and transmission time. Although the maximum radio transmit power is limited, the transmission time varies by applying different modulation and coding techniques, changing the data transmission speed over time. In conclusion, energy consumption is affected by the main three parameters for each data transmission process as follows:

1. The Packet Repetition Factor
2. The Radio Power
3. The Number of Retransmissions

### 2.5. Power Consumption Modeling for Data Processing

The simulations have considered a simple scenario, including different data processing algorithms using IoT device hardware which applies data compression techniques [17,18], to collect the data. Estimating the number of operations performed to do a specific task to calculate energy consumption is necessary to create a realistic simulation model. To estimate the consumed energy for all collected data, we need to calculate the number of operations by the required number of clock cycles to perform that operation using a particular hardware and processing unit.

### 2.6. Power Consumption Modeling of Data Acquisition

The power grid status in the smart grid can be estimated and observed by monitoring the collected data. Monitoring applications of power systems can be categorized into two main parts:

1. Monitoring regularly the power system which can happen periodically with a fixed time interval in between;
2. Monitoring power systems in an event-driven way, observing exceptional cases that happen randomly or due to alarms.

### 2.7. Modeling of Energy Consumption for NB-IoT according to 3GPP Standards

Essential requirements such as lifetime, available energy, and reduced cost need to be considered in modeling IoT applications energy consumption. For example, the power consumption of NB-IoT devices for a battery with 5Wh capacity and certain traffic conditions has been predicted in [5]. The assumption of the analysis is that UE periodically transmits a single data packet of a given size. For example, the battery life of UE, communicating 200-bytes of uplink data per day on average with this MCL can last for up to 10 years [5].

Our simulation using a Point-to-Point Communications (PPC) [15] model for a communication scenario with an Ideal MAC layer, an interference-free channel and a single hope data link. We worked on the 3GPP power consumption model, which is well understood by the research community and discussed in several papers. For example, in [19] the authors presented the first empirical NB-IoT power consumption model to measure the battery lifetime. According to this published paper, the power consumption in the first generation of NB-IoT devices is slightly higher than the 3GPP model. As a result, the authors measured a 10% shorter battery lifetime for this generation of NB-IoT hardware. We proposed D2D links and small cell deployment to improve the coverage for users in outage and increase the battery lifetime by reducing the required power to communicate to BS via a nearby device or small cell.

In addition, data compression techniques including Lempel-Ziv-Welch (LZW) and Huffman have been evaluated in our simulations and practical implementation for their processing time and compression performance.

(1) Lempel-Ziv-Welch (LZW): This compression method is an algorithm that taking advantage of symbol repetition to compress data [20]. It operates by creating a “dictionary” of symbols and associated codewords both for compression and decompression. The process of data size reduction in LZW is straightforward; it assigns a codeword for each string and using single codewords instead of repeated strings based on the primary dictionary, and adding new codewords to the existing dictionary with the unique reference number. Therefore, by compression of each new string, the LZW dictionary is updated with new codewords for incoming longer strings, and it replaces them with smaller codewords. By continuing to compress the data in this way, the LZW algorithm can compress data on the fly. LZW performs very well for compression of data sequences with repetitive substrings such as text and numeric files.

(2) Huffman (Huff): The basic principle of Huffman coding is to allocate bit patterns to characters according to their repetition frequency [21]. Therefore, two passes are required for compressing the file—one pass to find the rate of recurrence of each character and generate the Huffman tree and a second pass to actually compress the file. Huffman coding suffers from the fact that the decoder needs to have knowledge of the mapping of bit patterns to the characters. Sending this information with the codewords increases the overall bit rate. Conversely, if this information is unavailable, it will not be possible to decode the compressed data. In this practical implementation, for simplicity, we have implemented the Huffman compression technique. Still, in our other research work [17,19], to solve the problems associated with this technique, we have used an improved variant of Huffman coding called Adaptive Huffman (AH).

## 3. Simulation Results

In this section, simulation results from different communication scenarios and energy modeling approaches that have been discussed in the previous section will be described. Key simulation settings are shown in Table 2.

In the first step, our simulation analyzed how D2D communications assists the outage users communicate to the BS or a small cell via other users located within the coverage area of the macro-cell BS for the scenario in Figure 1. There are two issues in this scenario, the first one is the security of data transmissions with multi-hop communications between MTC devices and the second one is the increased energy consumption. Regardless of the security issue, which is not in the scope of this paper, we considered an energy efficient scenario where only one extra hop is allowed to extend the coverage to the users in outage. For the proposed model, the users in outage discover nearby MTC devices within the coverage area of the BS or small cells and measure the required energy to transmit the data packets to those devices. Then the network will choose the most energy efficient communication link to one of the nearby devices for data transmission.

**Figure 4:**
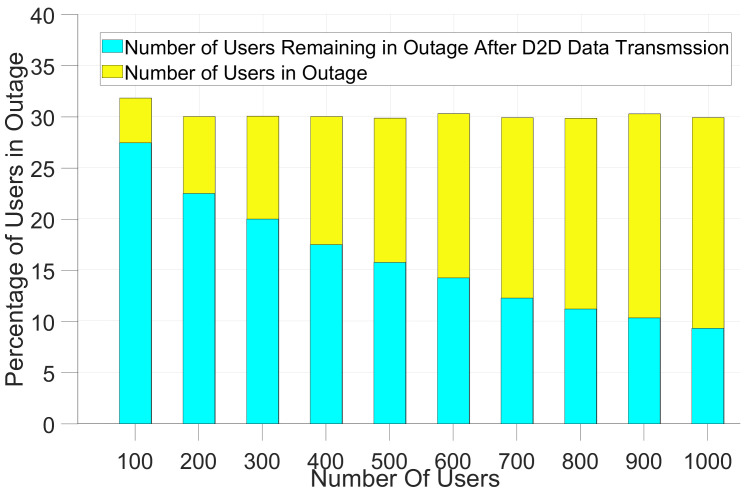
Device-to-Device communications to reduce the number of outage users.

Table 3 shows the baseline coverage analysis for both LTE and NB-IoT devices. Table 3 depicts that NB-IoT with an extra 20 dB of permitted MCL can reduce the percentage of users in outage to half of that for devices using LTE communication technology in the same size macrocell. As can be seen in Figure 4, the total percentage of outage users for NB-IoT is around 30% without deploying small cells or D2D. By increasing the number of MTC devices within the macro-cell coverage region, the proportion of outage users can be improved to 27% and to 10% with 100 and 1000 D2D enabled users, respectively. The result shows that D2D communication can also be considered an effective solution to improve coverage if the issues associated with energy consumption and security are resolved for MTC devices.

**Figure 5:**
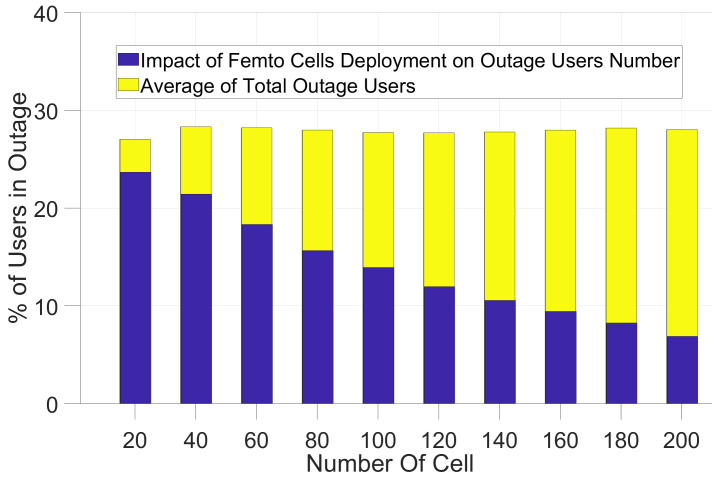
Simulation of macro-cell in the presence of Femto cells with Path-loss model—Equation (2).

**Figure 6:**
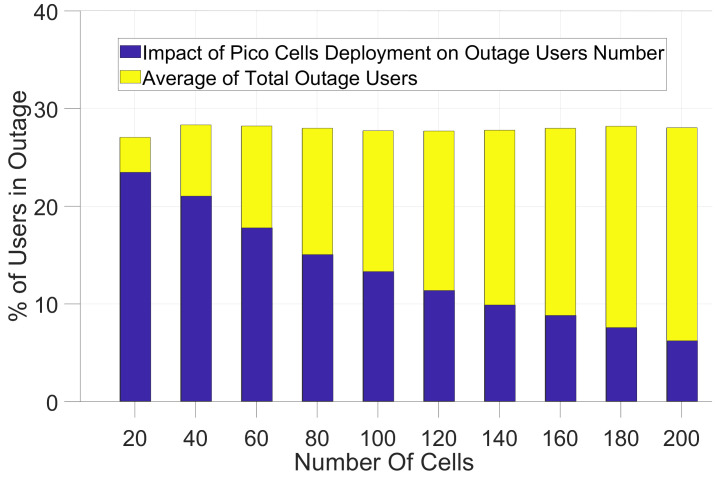
Simulation of macro-cell in the presence of Pico-cells with Path-loss model— Equation (2).

In Figure 5 and Figure 6 and Table 4, the results of the small cell communication scenario described in Figure 1 has been shown. Using the path-loss models and shadow-fading values for small cells, we simulated the coverage impact of pico-cells and femto cells. First, we analyzed the improvement of coverage for users in outage as can be seen in Figure 1 and Figure 2 for the path-loss model shown in Equations (2) and (3).

An interesting point is about how results differ from [3]. First of all, the number of outage users does not decrease significantly for a small number of 20 femto cells. Still, for both path-loss models in Figure 2, around 25% of users will remain in outage. On the other hand, by increasing the number of femto cells to 200 femto cells, we can improve the coverage and the number of outage users will be reduced significantly when compared to the simple model in [3]. The results in Figure 5 and Table 4 show that only 7.3% and 6.8% of users will remain in the outage for two path-loss models, respectively.

The results for deploying pico-cells are different. In contrast to [3] which has less than 15% of users in outage for deploying only 20 pico-cells, the results in Figure 6 and Table 4 do not show significant outage reduction with this number of pico-cells. This is regardless of their being inside the buildings or outside the building for a realistic shadow-fading map. By deploying many pico-cells (200 pico-cells), the percentage of outage users reduces to 7% and 6.1% of user devices in the macro-cell for the path-loss models.

**Figure 7:**
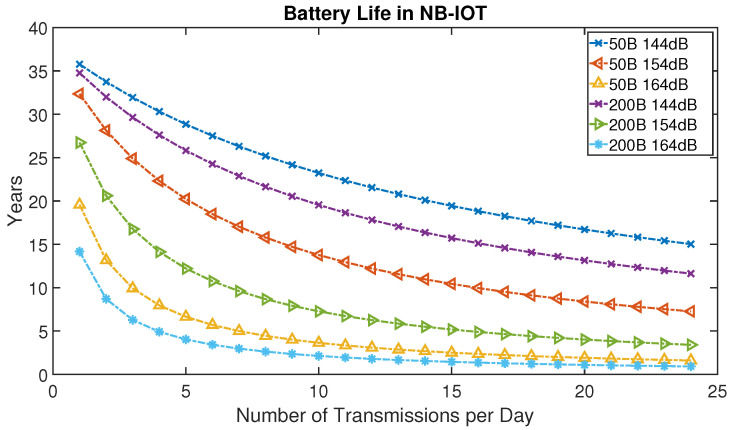
NB-IoT battery lifetime analysis for short data packets of 50 bytes (B) and 200 bytes (B) for Different MCL.

Using Equation (6), the energy consumption for data collection can be modeled using our simulator, based on how many samples have been collected by the acquisition hardware and the reporting requirements for the control center. As an example, in Figure 7 we analysed the NB-IoT battery lifetime for transmitting short data packets of 50 bytes and 200 bytes versus the number of reporting intervals per day for different values of the maximum coupling loss (MCL) based on [5]. From the figure it can be seen that battery lifetime for NB-IoT user devices can be increased by the shortening the data packets and by reducing the number of reporting times per day. For example, the lifetime of the battery for a single data transmission of 50 bytes per day with an MCL of 164 dB is 20 years, while the lifetime of the battery will reduce to 15 years for one transmission per day of 200 bytes with the same MCL. One way to shorten the data packets is using lossless compression techniques described in [17]. Results for this approach making use of a realistic testbed system based on Raspberry Pi computers is described in the next section.

Evaluation of the required energy consumption for data compression in real hardware is necessary to count the energy consumption of user devices which can significantly impact the battery lifetime of NB-IoT devices. Compression of NB-IoT data packets, in addition to increasing lifetime of the battery, can reduce the latency and increase reliability through using smaller data packets at a particular time. The energy consumption and compression of data is very important especially if the UE acts as a D2D node to extend the coverage of cellular IoT BS to outage users. By compressing data packets and the reducing the reporting interval to once or twice per day we can successfully increase the battery lifetime of MTC devices.

## 4. Testbed Results

In this section, we move on to discuss results from a smart grid experimental testbed. This uses a Laptop PC as a network controller and low-cost Raspberry Pi computers to emulate client-side devices that can implement advanced smart grid applications, such as demand response. This system makes use of the UK internet network to emulate a practical smart grid system.

### Impact of Compression on Cellular Communications Latency

One of the most critical parameters in smart grid communications is latency. Besides coverage analysis of NB-IoT technology, this new IoT technology’s latency characteristic is an essential factor in designing systems based on NB-IoT. As NB-IoT is not yet rolled out widely, we have tested the compression technique on the fourth generation (4G) and the third generation (3G) of cellular communication technologies in reality. In this paper, we have measured the one-way latency experimentally, which is defined as the time required for a data packet to be communicated from the transmitter to the receiver, including data compression if used.

It is worth mentioning that NB-IoT is based on the Long-Term Evolution(LTE) technology used in the 4G cellular network. Therefore, experiment related to 4G can provide a measure to evaluate the closely related technologies such as NB-IoT.

Data transmission in an IoT network has been emulated by creating a short data packet size from 50 bytes to 10 Kbytes which communicated from the client platform (Raspberry Pi 3B) to the server platform (Laptop PC) using 3G and 4G communication systems. Data sources in smart grid applications vary a lot, but for the purpose of demonstration the data used here was taken from the MIT Reference Energy Disaggregation Dataset (REDD) [22]. This dataset comprises a set of power consumption measurements from six houses, which is converted into energy consumption values recorded every 10 min—more details can be found in [17].

The impact of compression techniques on latency has been studied using two lossless compression algorithms, Huffman coding and Lemple–Ziv Welch(LZW). The performance of data reduction of two algorithms has been compared by calculating the space-saving ratio for those compression techniques as shown in Equation (8) and Table 5.

(8) $$\mathrm{Space}-\mathrm{Saving}\;\mathrm{Ratio}=1-\frac{\mathrm{Compressed}\;\mathrm{Data}}{\mathrm{Uncompressed}\;\mathrm{Data}}$$

It is essential that keep in mind by applying a compression algorithm while reducing the data size, it will increase the processing time both for compression and decompression of the data packet size, as is depicted in Equation (9).

(9) TotalLatency=CompressionTime + TransmissionLatency + DecompressionTime

Table 6, showing the compression and decompression processing time in a client platform (Raspberry Pi 3B+) for the selected lossless compression techniques. This type of processor is representative of what may be used in an advanced client device implementing sophisticated smart grid functions such as demand response [23]. In simpler devices such as smart meters, it is more common to use lower power microcontroller devices, which would require a longer processing time. Nonetheless, the relative comparison of the two methods would still be reasonable. The LZW and Huffman coding algorithm’s processing time is different on a hardware platform such as RPi as a client. Table 6 shows that the LZW compression time is much higher than the Huffman coding compression time and vice versa; the LZW decompression processing time is much less than that for the Huffman coding algorithm.

We need to keep in mind that the performance of the compression algorithm would change according to the type of data as discussed in [24]. As seen in [24], the Huffman algorithm can achieve a high compression ratio regardless of the data type considered, such as temperature data, humidity data, ECG data, and text files. At the same time, the LZW has poor performance on numerical data types such as temperature, humidity and ECG data, while it can perform better on compressing text files. The dataset we used in our work from [22] is an alphanumeric data type including date, time, circuit number and power consumption. For a server platform using a strong PC, the compression and decompression algorithm differences are not too much for both compression techniques. From Table 6, it can be predicted that using the Huffman algorithm on client platforms with weak hardware can be much more efficient than LZW. Based on the evaluation results described above, a 60–80% reduction in data packet size can be achieved with the Huffman coding algorithm which requires less than 20 ms processing time for data packet sizes up to 2 KBytes.

**Figure 8:**
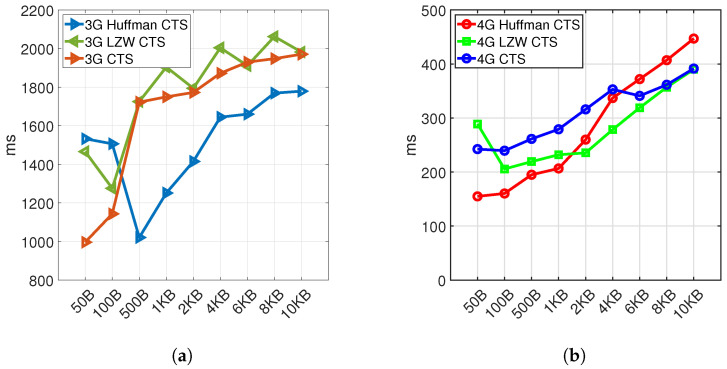
3G (**a**) and 4G (**b**) median latency without and with Huffman and LZW compression techniques (CTS stand for Client-to-Server (Uplink)).

**Figure 9:**
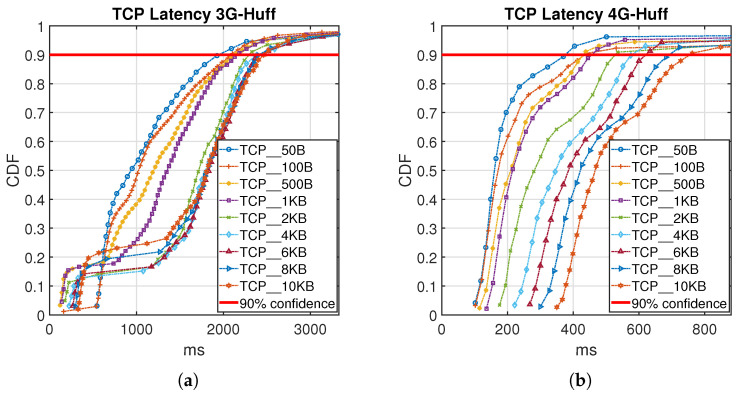
CDF plot for different data packet size both for 3G (**a**) and 4G (**b**) using Huffman Compression.

**Figure 10:**
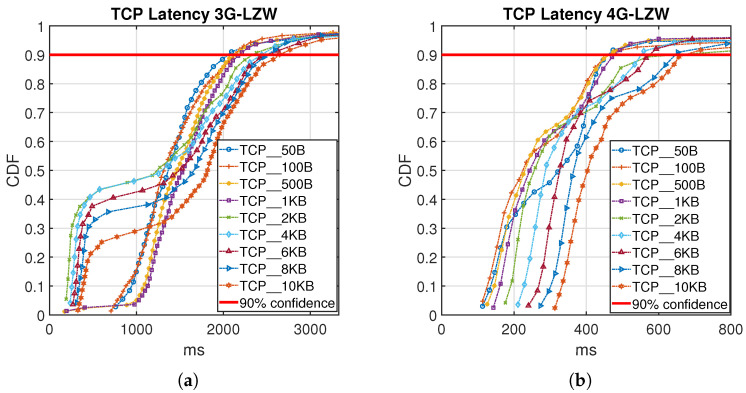
CDF plot for different data packet size both for 3G (**a**) and 4G (**b**) using LZW Compression.

In this research work, we have compared wireless last-mile communication technologies as shown in Table 7, based on estimated latency and data rate values that can be found from the literature and previous research work [23,25]. According to the references [23,26,27] MCL (signal strength) can impact significantly on the value of the latency. The latency for two standard protocols—the transmission control protocol (TCP) and user datagram protocol (UDP)—have been simulated for a smart grid IoT network in [28]. Our experimental results are for 3G and 4G links using a standard TCP implementation with Nagle’s algorithm activated. Results with and without compression techniques with different data packet sizes are illustrated in Figure 8, Figure 9 and Figure 10. Our prediction for NB-IoT is based on our experiments on 3G and 4G technologies.

This prediction has been proved from a practical experiment applying two compression algorithms on different data packet sizes shown in Figure 8. This figure shows the median latency value and compares latency measurements for different data packet sizes using the TCP protocol with and without applying compression techniques. Figure 8a,b show that using Huffman coding, especially for data packet sizes less than 4kbytes, are much more efficient than using LZW on the client side.

Figure 9 and Figure 10 show the Cumulative Distribution Function (CDF) of collected latency measurement from the testbed in detail for both 3G and 4G cellular network using LZW and Huffman coding algorithms. The red line plotted in the figures represents a 90% confidence latency value for the obtained results.

The 4G test TCP results in Figure 9 and Figure 10, for both Huffman coding and LZW shows more predictable behavior than the 3G results. It can be seen that Huffman coding generally provides a 10–20% lower latency than the LZW method and the uncoded case. Increasing the size of the data packet will increase the latency values. The very high latency results for 3G wireless technologies in Figure 9 and Figure 10 mainly is because of higher data packet loss that in detail has been presented in [23] for transmitted data without using compression techniques.

## 5. Conclusions

In this research work, different research questions have been answered using simulation and experimental approaches to increase the efficiency of future IoT technologies. This includes methods to improve coverage and reduce the probability of communications outage, increasing battery lifetime using compression techniques, and reducing latency. We proposed small cell deployment and D2D communications to improve coverage for UEs experiencing outage conditions and compression algorithms to improve communications efficiency. Thus, we could conclude the paper in two parts; simulation and empirical parts.

In the simulation section, we have studied coverage using different path-loss models, and realistic shadow-fading maps to evaluate cellular coverage for NB-IoT data services, a significant reduction in the proportion of outage users by deploying pico-cells—from 30% for no small cells to around 5–7% for 200 pico-cells—has been shown as one of the main results of this paper. Furthermore, we used a realistic power consumption model to study how energy consumption can be reduced by compressing data packets or reducing the reporting interval when using NB-IoT. Additionally, we proposed the Huffman compression technique to reduce the data volume and increase the battery lifetime of IoT devices. Moreover, we have analyzed the performance of NB-IoT for different smart grid applications as an LPWAN communication technology in terms of coverage area, data packets and the active number of smart meters in a Macro-cell.

Finally, in the experimental section, we have explored the characteristics of Huffman and LZW compression algorithms on 3G and 4G cellular communication technologies, and the impact of these compression algorithms on latency has been evaluated. It was found that Huffman coding generally performed better than LZW and could offer a modest reduction in communications latency of up to 10–20%. But for data packets close to the maximum transmit unit (MTU) in TCP, the Huffman performance will increase 30–40%. This better performance is because communicating one MTU in TCP protocol can be transmitted in a single network-layer transaction.

In future research, alternative compression methods need to be investigated, considering the impact of packet loss and errors on communication systems. Additionally, the realistic energy consumption of devices using LPWAN technologies (especially NB-IoT) need to be investigated considering joint compression and retransmission mechanisms to provide a high probability of successful transmission in the proposed communication architecture.

## Figures and Tables

**Table 1:** Communication Power consumption for NB-IoT model and devices ($E_{\mathrm{Communication}}$).

NB-IoT	3GPP Model [5]	Actual Devices [19]
Standby	0.015 mW	0.013–0.035 mW
Sleep	3 mW	21–23 mW
Transmit	480 mW	716–840 mW
Receive	75 mW	213–240 mW

**Table 2:** Simulation Key parameters.

Parameters	Values
NB-IoT Macro-cell Radius	6 km
Path-loss model Base Station-IoT Device distance: d(km)	Equations (3) and (4)
Log-normal fading standard deviation	from shadow-fading map
Femto eNB EIRP	20 dBm
Pico eNB EIRP	35 dBm
Maximum Transmit Power of MTC device	23 dBm
Maximum Transmit Power of Main BS	46 dBm
Number of Small Cells	Up to 200
Number of MTC Devices	300 and up to 1000 for the D2D scenario
$f_{c}$ for NB-IoT	900 MHz

**Table 3:** Comparison of the number of outage users in NB-IoT and LTE (4G) technologies .

Communication Technology	Percentage of Users in Outage
LTE	71
NB-IoT	28.6

**Table 4:** Results from Equations (3) and (4) for Femto and Pico-cells deployment and remaining users in Outage in each scenario.

No. of Small Cells	20	60	100	140	180	200
% Average Outage Users	28	28	28	28	28	28
Equation (3) Femto Cell	24.6	18.7	14.41	11.2	8.4	7.3
Equation (3) Pico-Cell	23.7	18.4	14.1	10.7	8.4	7
Equation (4) Femto Cell	23.6	18.3	13.9	10.5	8.2	6.8
Equation (4) Pico-Cell	23.4	17.7	13.2	9.8	7.5	6.1

**Table 5:** Percentage of Space saving.

Platform/Data Size	50 B	100 B	500 B	1 KB	2 KB	4 KB	6 KB	8 KB	10 KB
Huffman	55	65	77	79	80	80	80	80	80
LZW	−91	−46	−3	22	32	50	57	59	62

**Table 6:** Compression (ComT) and Decompression (DeComT) Time (ms).

Platform/Data Size	50 B	100 B	500 B	1 KB	2 KB	4 KB	6 KB	8 KB	10 KB
RPi (LZW-DeComT)	2	2	6	9	13	16	14	17	20
RPi (LZW-ComT)	3	5	13	24	42	75	91	100	106
RPi (Huff-DeComT)	2	2	11	24	47	78	89	101	116
RPi (Huff-ComT)	1	1	2	5	12	23	34	40	39

**Table 7:** Characteristics of 3GPP standardized wireless technologies used in the testbed.

	NB-IoT	4G	3G
Typical Latency	300 ms [27,29]-few seconds [27,30]	50 ms	100 ms
Data Rate (bps)	<150 K	15–50 M	1.5–8 M

## Data Availability

The software used for the experiments reported in this paper is available on request from the authors via email.
